# Role of NOD2 and hepcidin in inflammatory periapical periodontitis

**DOI:** 10.1186/s12903-022-02286-z

**Published:** 2022-06-28

**Authors:** Jing Hu, Marie Aimee Dusenge, Qin Ye, Ya-Qiong Zhao, Li Tan, Yao Feng, Jie Zhao, Zheng-Rong Gao, Shao-Hui Zhang, Yun Chen, Ying-Hui Zhou, Yue Guo, Yun-Zhi Feng

**Affiliations:** 1 Department of Stomatology, The Second Xiangya Hospital of Central South University, Changsha, 410011 Hunan China; 2 National Clinical Research Center for Metabolic Diseases, Hunan Provincial Key Laboratory of Metabolic Bone Diseases, and Department of Metabolism and Endocrinology, The Second Xiangya Hospital of Central South University, Changsha, 410011 Hunan China

**Keywords:** Periapical periodontitis, NOD2, Hepcidin, Inflammation

## Abstract

The immunological response occurring during periapical inflammation includes expression of nucleotide binding oligomerization domain containing 2 and hepcidin. Nucleotide binding oligomerization domain containing 2 deficiency increases infiltration of inflammatory cells close to alveolar bone. Hepcidin has an important role in iron metabolism affecting bone metabolism.We investigated the role of nucleotide binding oligomerization domain containing 2 and hepcidin in inflammatory periapical periodontitis. Periapical periodontitis was induced in rats and confirmed by micro-computed tomography. Nucleotide binding oligomerization domain 2 and hepcidin were evaluated through immunohistochemistry. Bioinformatics analysis was undertaken usingthe Kyoto Encyclopedia of Genes and Genomes and Gene Ontology databases. Micro-computer tomography revealed alveolar bone resorption in the periapical region and furcation area of mandibular molars in rats of the periapical periodontitis group. Immunohistochemistry showed increased expressionof nucleotide binding oligomerization domain containing 2 and hepcidin around root apices in rats of the periapical periodontitis group. Bioinformatics analysis of differentially expressed genes in inflamed and non-inflamed tissues revealed enrichment in the NOD-like receptor signaling pathway. Our data suggest that nucleotide binding oligomization domain contain2 and hepcidin have important roles in periapical periodontitis severity because they can reduce alveolar bone loss.They could elicit new perspectives for development of novel strategies for periapical periodontitis treatment.

**Supplementary Information:**

The online version contains supplementary material available at 10.1186/s12903-022-02286-z.

## Introduction

A periapical lesion involves an inflammatory process affecting the soft and hard tissues surrounding the tooth. Periapical periodontitis (PP) is an inflammatory disease of periradicular tissues. It occurs in response to infection of dental pulp due to trauma, dental caries, or iatrogenic factors [1]. PP develops from a complex inflammatory immune response triggered by microbial elements which, ultimately results in bone destruction [2].

Various proinflammatory mediators have important roles in the occurrence and development of PP and the reaction that induces bone resorption. Interleukin (IL)-6 and IL-8 enhance infiltration of inflammatory cells and promotes bone remodeling. Similarly, tumor necrosis factor (TNF)-α helps the initiation and regulation of the inflammatory process through activation and differentiation of osteoclasts and collagen production [3–5].

Nucleotide binding oligomerization domain containing 2(NOD2) is a protein that can recognize different peptides in bacterial walls. NOD2 mediates activation of nuclear factor-kappa B (NF-κB) and expression of the proinflammatory cytokine TNF-α, thereby initiating the immune response to pathogens. It has been suggested that NOD2 deficiency increases the infiltration of inflammatory cells near alveolar bone [6]. In addition, a balanced NOD2 signal is essential for maintaining homeostasis of the immune system. An inactive form of NOD2 (caused by NOD2 mutation) or high expression of NOD2 is associated with various inflammatory diseases [7]. Therefore, the role of NOD2 in PP is not clear, and further research is needed.

Ubiquitin, methylation, and endoplasmic reticulum stresscan regulate various biological processes, including bone remodeling [8–10]. Iron overload regulated by hepcidin (a peptide hormone made in the liver) is also closely related to bone remodeling. Hepcidin is an iron regulating hormone that is primarily responsible for maintaining iron homeostasis. It causes degradation of the iron export protein ferroportin, which is necessary for iron transfer from enterocytes and macrophages to the systemic circulation. Increased iron stores cause hepcidin expression to be upregulated. Low hepcidin, on the other hand, boosts iron absorption from the gut, restoring iron stores [11, 12]. Hasler P and colleagues [13] showed that mice lacking hepcidin had a low-bone-mass phenotype, which may have been due to the decreased differentiation and activity of osteoblasts causing the inhibition of bone formation. The “vicious circle "between hepcidin deficiency and iron overload enhanced bone abnormalities in hepcidin-deficient mice [13, 14].It has been reported that, in the oral cavity, an increased serum level of iron increases the susceptibility to periodontal disease [15]. Ferritin, a protein that stores iron, was also found to be upregulated in periodontitis; factors like P.gingivalis-LPS, IL-6, and TNF- α, which are elevated in patients with periodontitis were confirmed to induce ferritin expression and secretion, suggesting the role of ferritin in development of periodontitis [16]. Patients with iron overload have been found to suffer from periodontitis [17].Whether hepcidin plays an important part in PPis not known.

We investigated the role of NOD2 and hepcidin in periapical inflammationaffecting bone remodeling in PP. We hypothesized that NOD2 and hepcidin have important roles in PPprogression.

## Materials and methods

### Ethical approval of the study protocol

The Ethics Committee of Second Xiangya Hospital of Central South University (Furong, China) approved (2021031) the study protocol.

### Experimental animal model

Ten male Sprague–Dawley rats (400 g; SJA Laboratory Animals, Hunan, China) were divided into two groups of five: control and PP.

### PP induction

PP was induced in rats in the PP group. First, anesthesia was injected (1% sodium pentobarbital, i.p.). In each rat, the pulp chambers of both sides of mandibular first molars were opened. Then, the pulp was removed from the pulp cavity. Exposure of the pulp chamber and pulp removal followed the guidelines set in *The Guide to Clinical Endodontics* published by the American Association of Endodontists [18, 19]. The pulp cavity was left open to the oral-cavity environment for 4 weeks to allow formation of a periapical lesion. Rats in both groups were given soft food to prevent possible tooth pain due to eating hard food. Micro-computed tomography (micro-CT) was undertaken 4 weeks later to observe the periapical condition of the first mandibular molars on both sides to confirm that the model had been created.

### Micro-CT

The right and left lower mandibles were removed and fixed in 4% paraformaldehyde (dissolved in phosphate-buffered saline) for 48 h. They were preserved in70% ethanol solution at 4°C until use. Three-dimensional projection images were reconstructed from a stack of two-dimensional images using a micro-CT machine (μ-CT50; Scanco, Basserdorf, Switzerland). Periapical lesions were imaged at 90 kV and 160 μA. Then, the images obtained from micro-CT were reconstructed using VGStudioMAX3.0 (Volume Graphics, Berlin, Germany). The low-density space around the mesial root of mandibular molars was measured and considered to be the volume of the periapical lesion.

### Immunohistochemistry

After the killing of rats, samples were obtained and embeddedin paraffin blocks. Immunohistochemistry was undertaken using the streptavidin–biotin method.Briefly, paraffin-embedded tissues were sectioned into slides at a thickness of 4 μm, dewaxed, and rehydrated in a graded series of ethanol solutions (100, 95, and 80%). Slides were heated for 30 min at 65 °C for antigen retrieval and left to cool naturally. After blockade with 5% bovine serum albumin (Beyotime Institute of Biotechnology, Shanghai, China) for 1 h at 37 °C, slides were incubated with antibodies against NOD2 (1:400 dilution; catalog number, NB100-524SSS;NovusBio, Littleton, CO, USA) and hepcidin (1:200; ab30760; Abcam, Cambridge, UK) for < 12 h at 4 °C. Antibody diluents were directly added to negative control. The next day, slides were washed thrice with bovine serum albumin and allowed to incubate with 50–100 μL of horseradish peroxidase-labeled secondary antibody for 30 min at room temperature. For the negative control each slide was treated with 50–100μLDAB working solution per tissue block, while the experimental group can be seen brown to the naked eye, each slide was cleaned with tap water and distilled water respectively. Then, dehydration was carried out with a graded series of ethanol solutions (80, 95, and 100%) for 2 s each time. Finally, the tissue sections were fixed with neutral gum, and the positive staining in the tissue was observed under a microscope (Olympus CX31) after fixation.

### Analyses of enrichment of function and signaling pathways of differentially expressed genes (DEGs)

Gene expressions in different inflamed tissues and uninflamed tissueswere compared based on public Gene Expression Omnibus (GEO) datasets (www.ncbi.nlm.nih.gov/gds) (Additional file 2: Fig. S1) and fifteen DEGs (fold change > 2, *P* < 0.05) were obtained through comparing different inflamed samples and uninflamed samples. Then, fifteen DEGs were mapped to GO and KEGG databases. Enrichment of function and signaling pathways was analyzed using the Gene Ontology (GO; www.genome.jp/kegg/), Kyoto Encyclopedia of Genes and Genomes (http://geneontology.org/) databases, respectively, using cluster Profiler (https://bioconductor.org/).

### Statistical analyses

Results are the mean ± SD. Statistical analyses were undertaken using Prism 8(GraphPad, San Diego, CA, USA) and Image J 1.49 m (Wayne Rasband, National Institute of Mental Health, USA). Analysis of variance was used to evaluate differences between the twogroups. *P* < 0.05 was considered significant.

## Results

### PP development

**Fig. 1:**
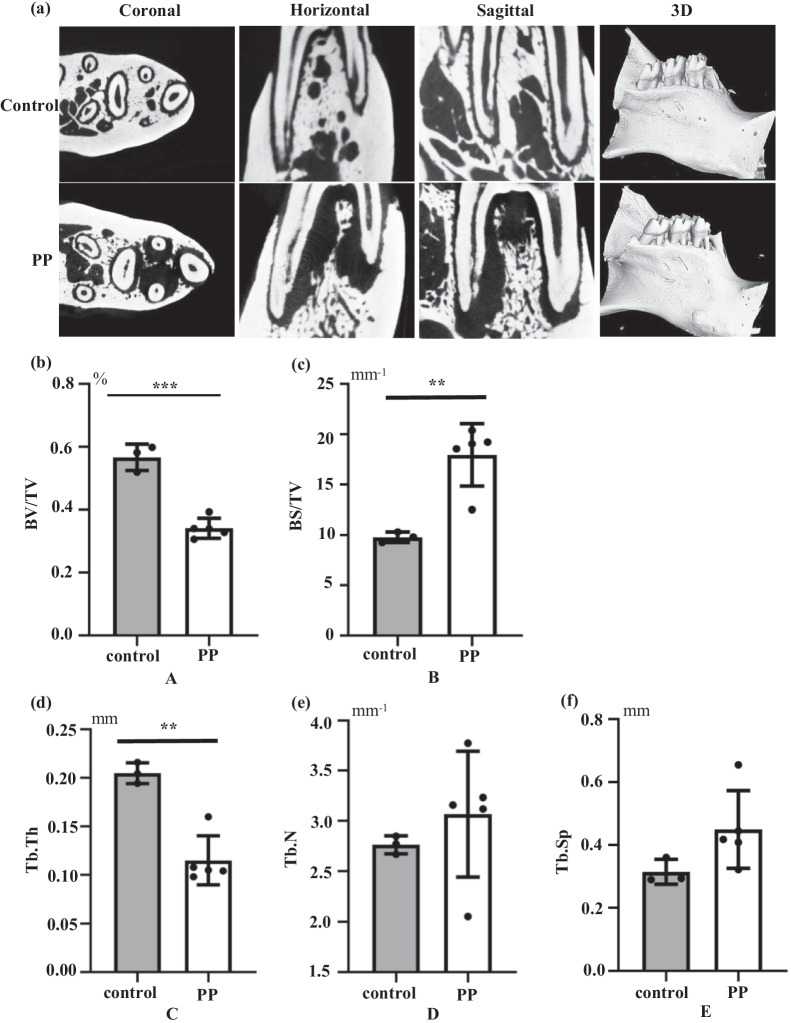
Alveolar bone resorption in control group and periapical periodontitis (PP) group. **a** Representative 2D images from coronal, horizontal, sagittal directions and 3D reconstruction of micro-CT of the mandible. **b** and **c** Quantitative analyses of the residual mandibular alveolar bone. Microstructural parameters of the trabecular bone in the mandible, including **d** trabecular thickness (Tb. Th), **e** number of trabecular bone (Tb. N), **f** trabecular bone clearance (Tb. Sp). *represents *P* < 0.05, **represents *P* < 0.01, ***represents *P* < 0.001. The data are the average ± SD

Bone resorption was identified and quantified by micro-CT. Radiolucent periapical regions indicated areas where the hard bone tissue had become a soft periapical lesion due to the inflammatory process Fig. 1a. Micro-CT of mandibular alveolar bone revealed that the trabecular bone volume (BV/BT) in the furcation area of mandibular first molars had undergone bone resorption. Representative micro-CT images ofa treated tooth compared with a control tooth are shown in Fig. 1b and c. A significantdifference between the two groups was noted for trabecular thickness (Tb. Th) in the mandible Fig. 1d. However, there was no significant difference in the trabecular number (Tb. N) Fig. 1e or trabecular separation (Tb. Sp) between the two groups Fig. 1f.

### NOD2 and hepcidinhavehigh expression in periapical tissues compared with that in controls

**Fig. 2:**
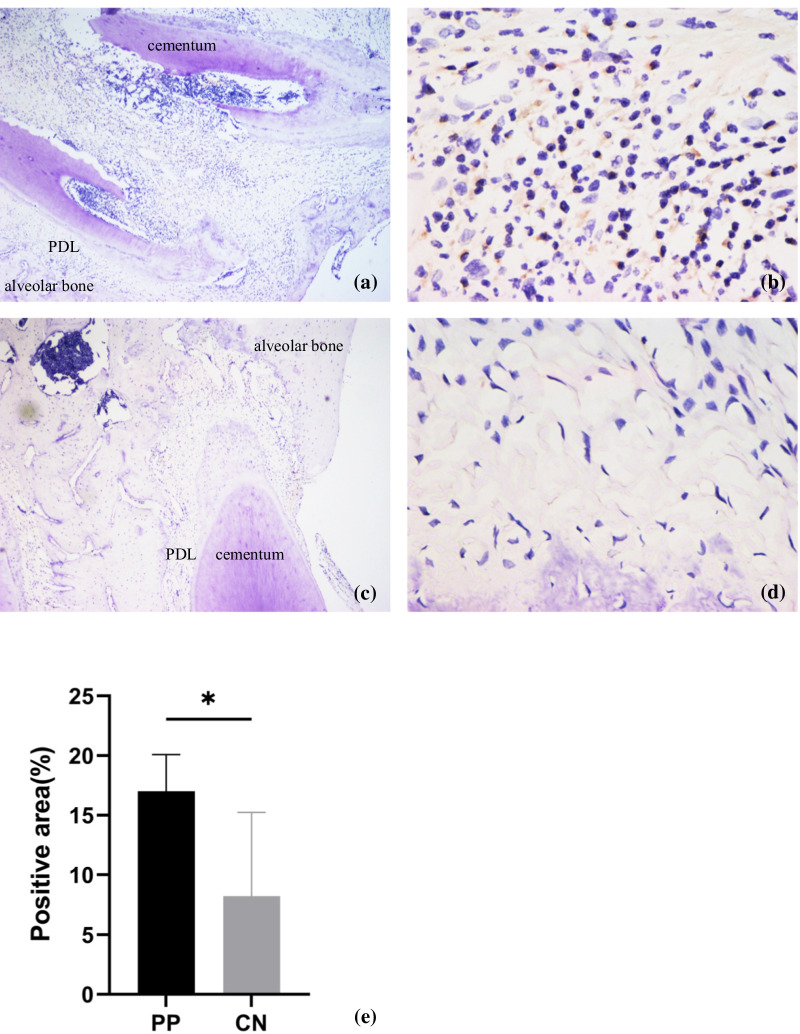
Immunohistochemical expression of NOD2 in periapical tissues. **a–d** Representative images of the NOD2-positive staining in induced rat periapical inflammatory lesions. Rat periapical lesions showed a negative observation of NOD2 in control group. **e** Quantitative analysis of the NOD2 expression.Original magnification: ×4 (**a** and **c**), ×40 (**b** and **d**)

**Fig. 3:**
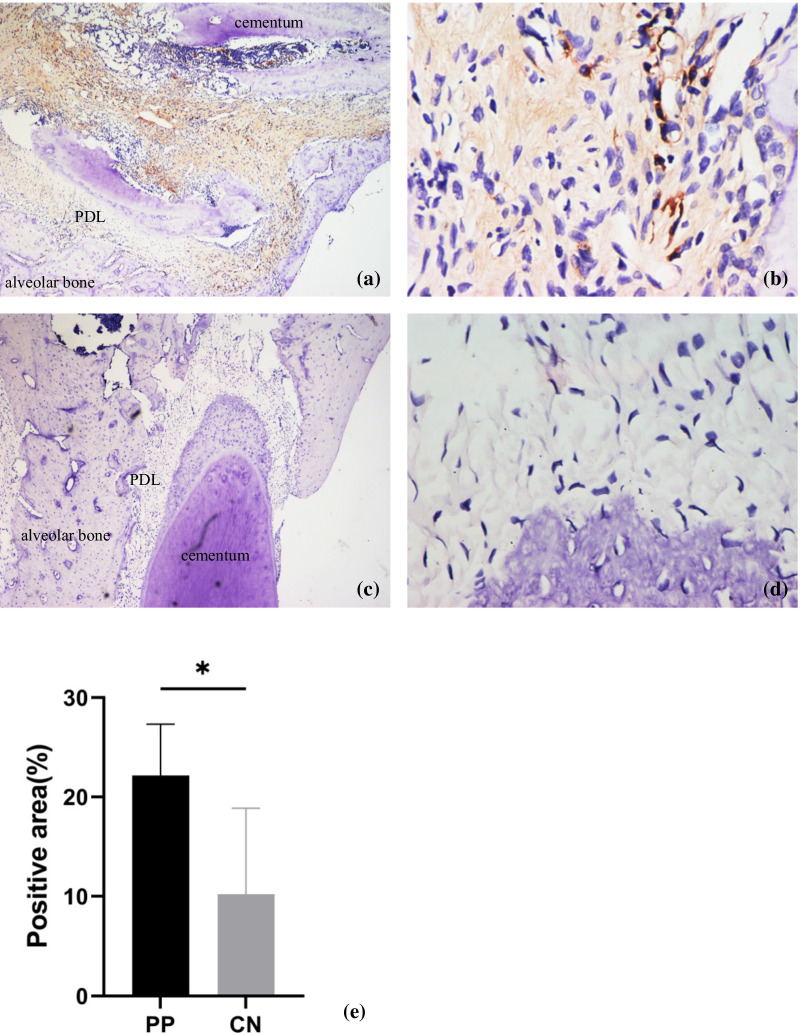
Representative images of the immunohistochemical expression of hepcidin in periapical tissue. Hepcidin-positive staining could be observed in induced rat periapical inflammatory lesions. Rat periapical tissue showed a negative observation of hepcidin in control group. Hepcidin was expressed on the ECM in periapical lesions. **e** Quantitative analysis of the hepcidin expression. Original magnification: ×4 (**a** and **c**), ×40 (**b** and **d**)

We analyzed cell infiltration by immunohistochemistry for further illustration of the inflammatory response in the area of interest. Figure 2 demonstrates NOD2 staining in the periapical tissues of mandibular molars of rats in the two groups. NOD2-positive staining was observed in induced periapical inflammatory lesions.NOD2-postive staining area in PP group is significantly larger than that of control group.Hepcidin staining in the periapical tissues of mandibular molars of rats between the two groups is shown in Fig. 3. Hepcidin-positive staining was observed in rats with induced periapical inflammatory lesions. Ratsin the control group with no periapical tissues did not show positive staining for hepcidin. Hepcidin was expressed on the extracellular membrane in periapical lesions.

### Enrichment in NOD-like receptor signaling of DEGs using KEGG and GO databases

**Fig. 4:**
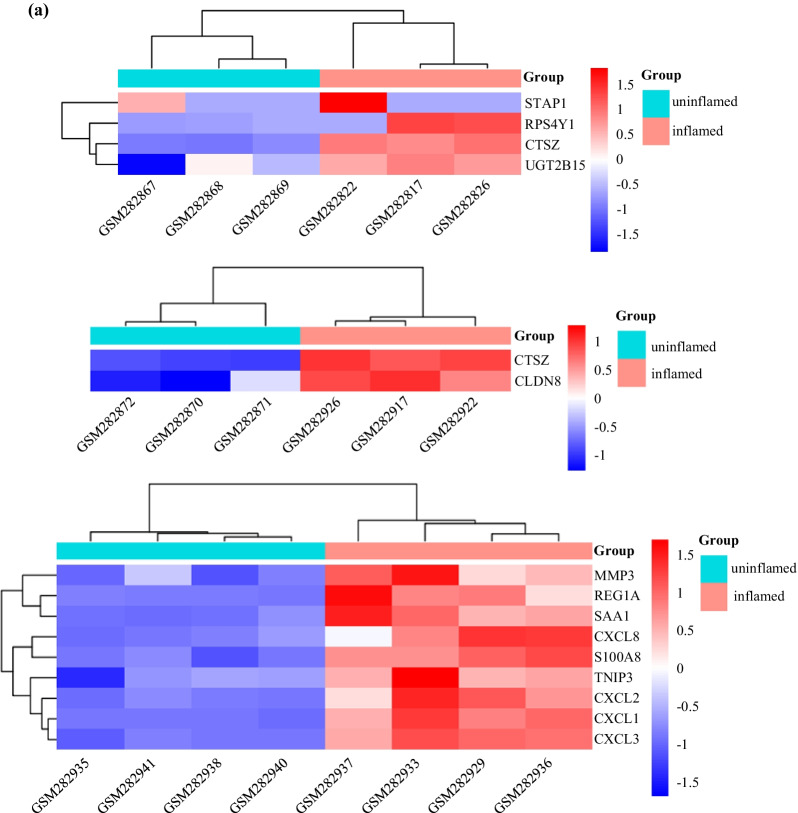

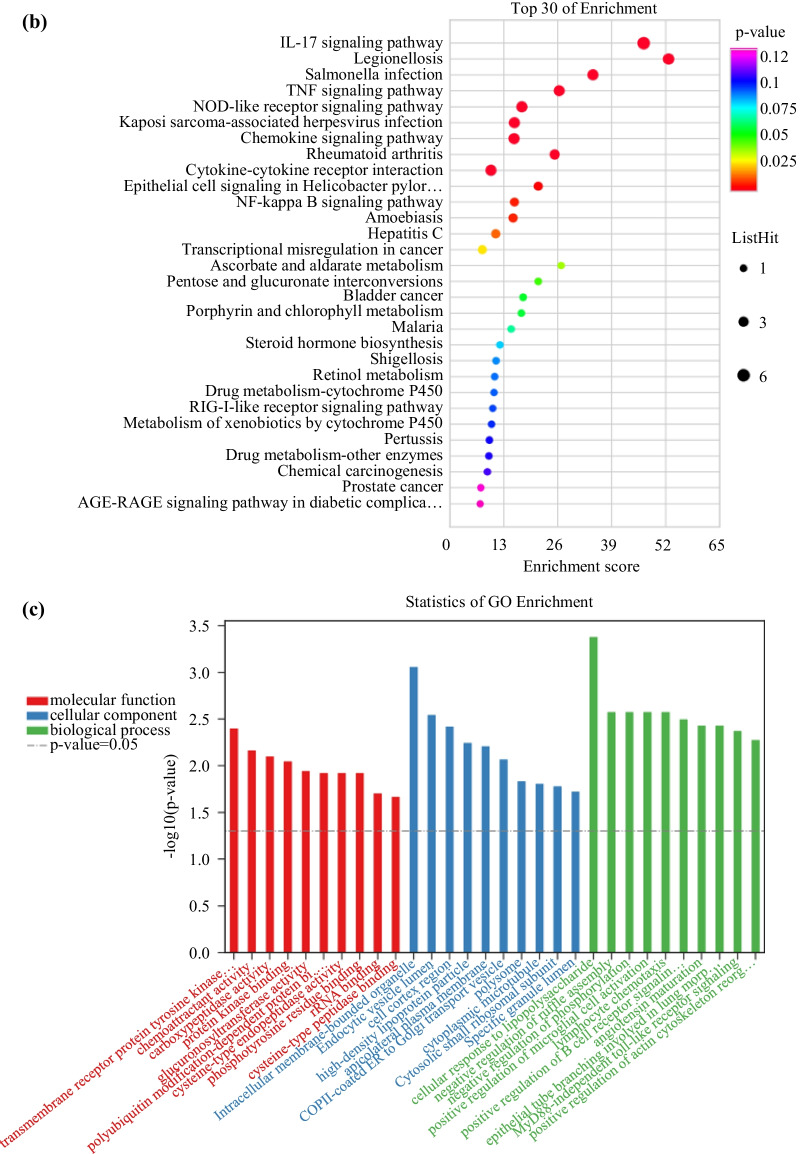
DEGs in inflammatory tissues were screened from GEO database for bioinformatics analysis. **a** Heatmaps of the differentially expressed genes with twofold higher the expression in the inflammatory sample as in the non-inflammatory sample. Red stripes represent high expression genes; blue stripes represent low expression genes. GSM represents sample from GEO Datasets. GSM represents sample from GSE4139 and GSE11223 Datasets. **b** Top 30 significant enriched KEGG pathway of 15 DEGs. **c** Top 10 significant enriched Gene Ontology (GO) including molecular function, cellular component and biological of 15 DEGs

The GEO data base identified the following DEGs in inflamed tissue and non-inflamed tissue (Fig. 4a), (Additional file 3: Table S1, Additional file 4: Table S2, Additional file 5: Table S3): Signal transducing adaptor family member1 (STAP1), ribosomal protein S4,Y-linked1(RPS4Y1), cathepsin Z(CTSZ), UDP glucuronosyltransferase 2 family polypeptide B15(UGT2B15), claudin8(CLDN8), matrix metallopeptidase3(MMP3), regenerating islet-derived 1 alpha (REG1A), serum amyloid A1(SAA1), chemokine (C-X-C motif) ligand 8(CXCL8), S100 calcium binding protein A8(S100A8), TNFAIP3 interacting protein3(TNIP3), chemokine(C-X-C motif) ligand 2(CXCL2), chemokine (C-X-C motif) ligand 1(CXCL1) and chemokine (C-X-C motif) ligand 3(CXCL3.). Figure 4b shows the top30 significantly enriched signaling pathways in these DEGs using the KEGG database, including the NOD-like receptor signaling pathway, IL-17 signaling pathway, and TNF pathway. Figure 4c shows the top10 significantly enriched functions of 15 DEGs using the GO database. For the molecular function (MF) classification, “trans-membrane receptor protein tyrosine kinase”, “chemoattractant activity” and “carboxypeptidase activity” were documented. For the classification of cellularcomponent (CC), the enriched functions were “intracellularmembrane-bounded organelle”, “endocytic vesicle lumen” and “cell cortex region”. For the biological process (BP) classification, the functions enriched were “cellular response to lipopolysaccharide”, “negative regulation of ruffle assembly”, and “negative regulation of phosphorylation”.

## Discussion

We established a model in rats to study the role of hepcidin and NOD2 in PP. After exposing the pulp cavity of the mandibular first molar for 4 weeks, the condition of periapical alveolar bone was evaluated by micro-CT to confirm establishment of the PP model. Immunohistochemical analyses were carried out to measure expression of NOD2 and hepcidin.The GEO database was employed to obtain DEGs in inflamed tissues and non-inflamed tissues. Analyses of functional enrichment and signaling-pathways enrichment using the GO and KEGG databases, respectively, was carried out to ascertain the effects of NOD2 and hepcidin in PP.

PP is a continuation of dental-pulp infection. It triggers a local chronic inflammatory immune response and impairment of periapical tissues, including the periodontal ligament, cementum, and alveolar bone [20]. NOD2 staining was positive in the periapical tissue of the mandibular first molar of rats suffering from PP, but not in the periapical tissue of the mandibular first molar of rats not suffering from PP. Analyses of signaling-pathway enrichment using the KEGG database showed that DEGs in inflamed tissues were enriched in the NOD-like receptor signaling pathway.

NOD2 is related to progression of the inflammatory response [7]. NOD2 recognizes the peptidoglycan components of bacteria, drives activation of mitogen-activated protein kinase and NF-κB pathways, leads to production of proinflammatory cytokines, and plays a critical partin protecting the body from pathogen invasion [21]. NOD2 is involved in recognizing certain bacteria and stimulating the immune system to respond appropriatelyto reduce the risk of bacterial infections [22] It has been reported that NOD2 expression is increased in areas of inflammation in rheumatoid arthritis [23] and atherosclerosis [24]. NOD2 promotes the progression of vascular inflammation by mediating the production of proinflammatory factors such as IL-8 [25]. In the oral cavity, activation of NOD2 by muramyl dipeptide (MDP) can upregulate expression of proinflammatory mediators and cytokines, thereby enhancing the immune response of dental pulp to pathogens [26]. *Porphyromonas gingivalis*is responsible for destruction of cementum and progression of PP in general [27, 28].Upregulation of NOD2 expression may be due to NOD2 activation by MDP in *P. gingivalis*, which participates in the progression of periapical inflammation. Analyses of functional enrichment of DEGs using the GO database indicated that DEGs were enriched mainly in regulation of the cellular response to lipopolysaccharide (BP), transmembrane receptor protein tyrosine kinase (MF), and intracellular membrane-bounded organelle (CC). GO enrichment was also associated with inflammatory progression.

Inflammatory progression is closely related to bone loss. Yuan and colleagues showed thatNOD2-deficiency in rats led to aggravation of inflammatory processes associated with atherosclerosis and periodontitis. NOD2-deficient ratshad increased numbers of resorbing osteoclasts, which supports the notion that NOD2 prevents bacteria-induced bone loss due to inflammation.They also showed that, the activation of NOD2 by MDP treatment in rats, result in a significant decrease in plaque accumulation, alveolar bone loss, and serum levels of cytokines and cholesterol [29]. Souza and colleagues induced periodontitis in rats and showed that NOD2 reduced bone resorption and osteoclastogenesis, but the reduction in bone resorption did not affect inflammation as observed by histology [30]. In our study, micro-CT showed root absorption and alveolar bone loss of the first molar of rats with PP.

Similar to changes in NOD2 expression in PP, hepcidin levels in inflammatory periapical lesions were higher than those in normal tissues. Several studies have demonstrated the role of hepcidin in reducing bone loss and preventing osteoporosis. Hepcidin deficiency might cause bone loss by interfering with the canonical wingless type/β-catenin pathway via Forkhead box-3a [31].

Hepcidin synthesis is controlled mainly by transcription. The primary systemic regulators of hepcidin include plasma iron concentrations, through the interaction of diferric transferrin with transferrin receptors TFR1 and TFR2 in the liver, hepatic iron stores, systemic inflammation, primarily conveyed to hepatocytes by IL-6, and erythroid activity. Anemia and hypoxia are the significant causes of hepcidin downregulation [32, 33].Hepcidin deficiency increases circulating levels of iron and leads to severe bone loss in rats [13]. Those results are not dissimilar to our findings; we revealed an increase of alveolar bone loss in rats of the PP group compared with that in the control group. Shen GS and collaborators showed that hepcidin deficiency inhibited an increase in the hepcidin level in response to iron accumulation, and caused severe iron overload in tissues, including iron overload in bone that affected the micro-architecture of bone [34]. Dissimilar results were reported by Guo and colleagues they suggested a negative role for hepcidin in regulation of bone homeostasis by promotion of proliferation and differentiation of osteoclast precursors. They implicated hepcidin in osteoblastic amyloid protein-induced osteoclastogenesis, and suggested that increased levels of hepcidin contributed to trabecular bone loss [35]. Hepcidin is associated with bone resorption in various diseases, and the hepcidin level was increased in the PP group in our study. Hence, we speculated that hepcidin plays an important partin the progression of alveolar bone resorption in PP. Analyses of signaling-pathway enrichment of DEGs using the KEGG database in the present study showed that hepcidin has an important role in PP progression. We found that DEGs were enriched in the NOD-like receptor signaling pathways well as other pathways. Fan and colleagues demonstrated that the gene for signal transducer and activator of transcription (STAT1) takes part in the gene–gene interaction network for hepcidin [36]. STAT1 is part of the NOD signaling pathway network, and we showed that the NOD-like receptor signaling pathway plays a partin PP progression. It suggested that there may be a connection between NOD2 and hepcidin, but whether there is a connection between NOD2 and hepcidin still needs further research.

## Conclusions

NOD2 and hepcidin have important roles in PP severity because they can reduce alveolar bone loss. They could elicit new perspectives for development of novel strategies for PP treatment.

## Supplementary Information

**Additional file 1**. Raw data.

**Additional file 2: Fig. S1** Top 30 significant enriched Gene Ontology (GO) including molecular function, cellular component and biological of 15 DEGs.

**Additional file 3**. Gene expression values of STAP1, RPS4Y1, CTSZ, and UGT2B15 in inflamed and non-inflamed tissues obtained from GEO datasets.

**Additional file 4**. Gene expression values of CTSZ and CLDN8 in inflamed and non-inflamed tissues obtained from GEO datasets.

**Additional file 5**. Gene expression values of CXCL8, REG1A, S100A8, SAA1, TNIP3, MMP3, CXCL1, CXCL2, and CXCL3 in inflamed and non-inflamed tissues obtained from GEO datasets.

## Data Availability

All datasets generated and analyzed during the current study are available under the supplements material section (Additional file 1).
