# Supplementary material for: Role of NOD2 and hepcidin in inflammatory periapical periodontitis
Source: BMC Oral Health. 2022 Jun 28;22:263. doi: 10.1186/s12903-022-02286-z (PMC9241313; doi:10.1186/s12903-022-02286-z)
Supplement: Supplementary file 1 — Additional file 1. Raw data. [file 12903_2022_2286_MOESM1_ESM.zip › Raw data/Raw data.pptx]

## Slide 1
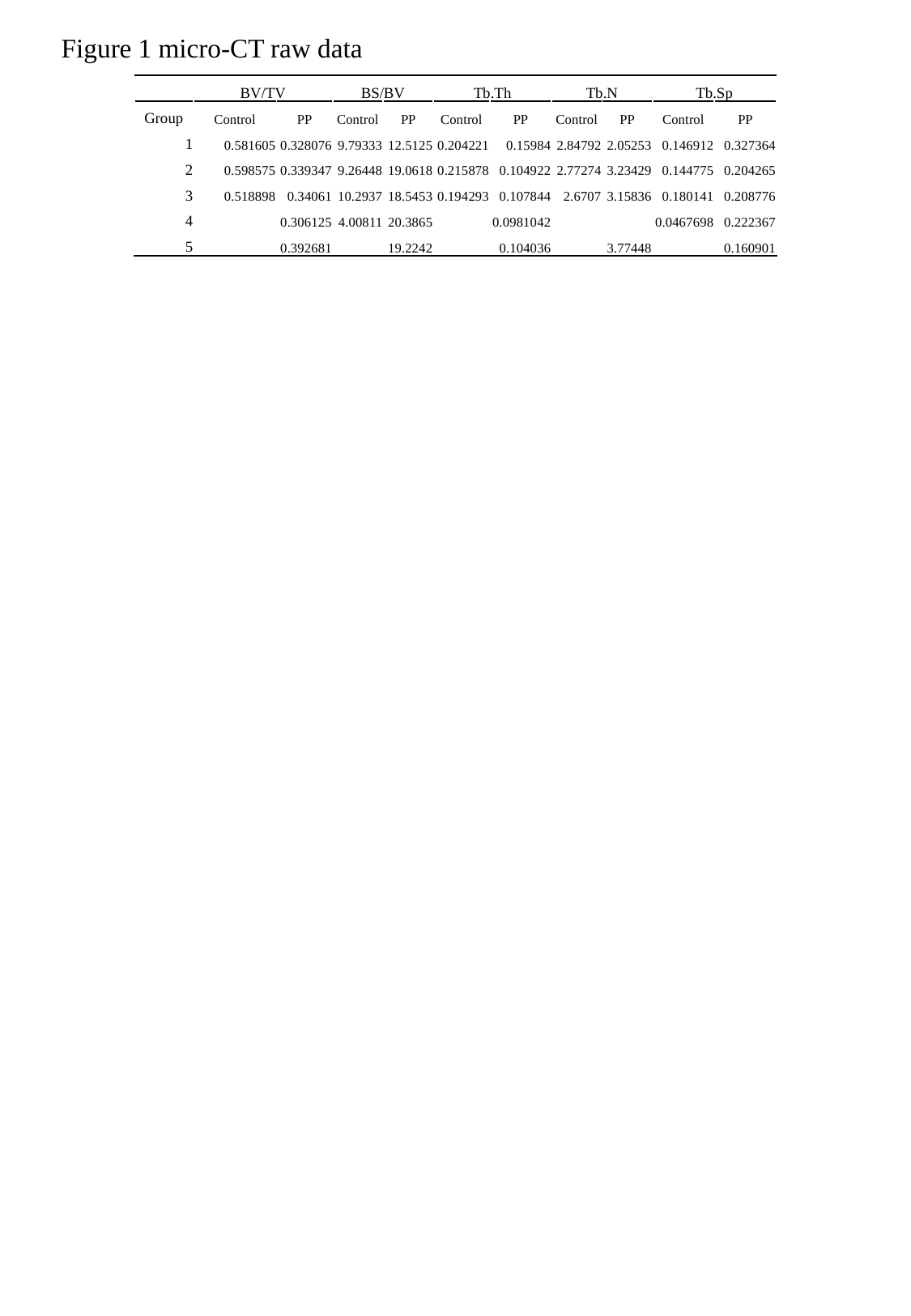

Figure 1 micro-CT raw data
| | BV/TV | | BS/BV | | Tb.Th | | Tb.N | | Tb.Sp | |
| --- | --- | --- | --- | --- | --- | --- | --- | --- | --- | --- |
| Group | Control | PP | Control | PP | Control | PP | Control | PP | Control | PP |
| 1 | 0.581605 | 0.328076 | 9.79333 | 12.5125 | 0.204221 | 0.15984 | 2.84792 | 2.05253 | 0.146912 | 0.327364 |
| 2 | 0.598575 | 0.339347 | 9.26448 | 19.0618 | 0.215878 | 0.104922 | 2.77274 | 3.23429 | 0.144775 | 0.204265 |
| 3 | 0.518898 | 0.34061 | 10.2937 | 18.5453 | 0.194293 | 0.107844 | 2.6707 | 3.15836 | 0.180141 | 0.208776 |
| 4 | | 0.306125 | 4.00811 | 20.3865 | | 0.0981042 | | | 0.0467698 | 0.222367 |
| 5 | | 0.392681 | | 19.2242 | | 0.104036 | | 3.77448 | | 0.160901 |

## Slide 2
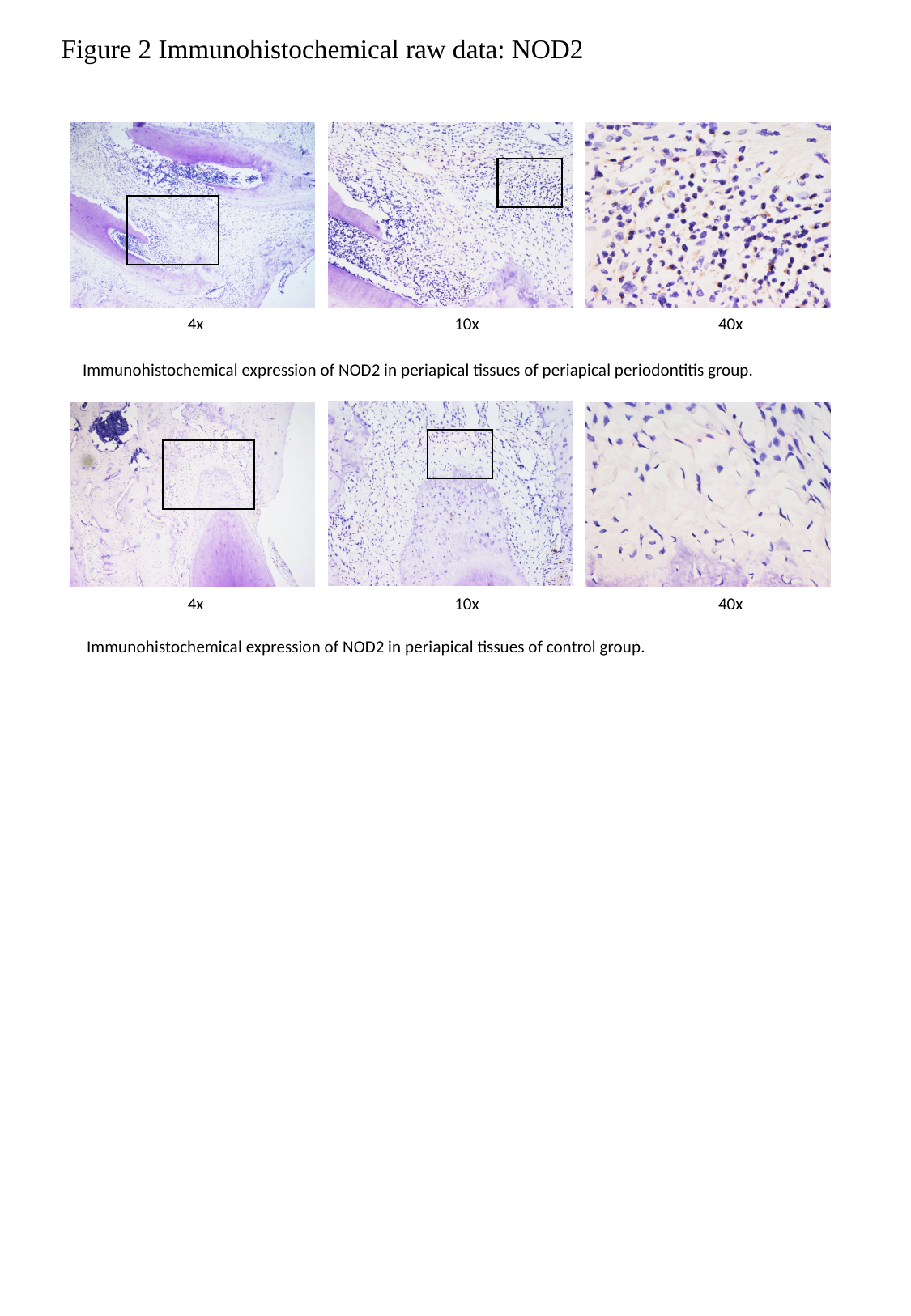

Figure 2 Immunohistochemical raw data: NOD2
4x
10x
40x
Immunohistochemical expression of NOD2 in periapical tissues of periapical periodontitis group.
4x
10x
40x
Immunohistochemical expression of NOD2 in periapical tissues of control group.

## Slide 3
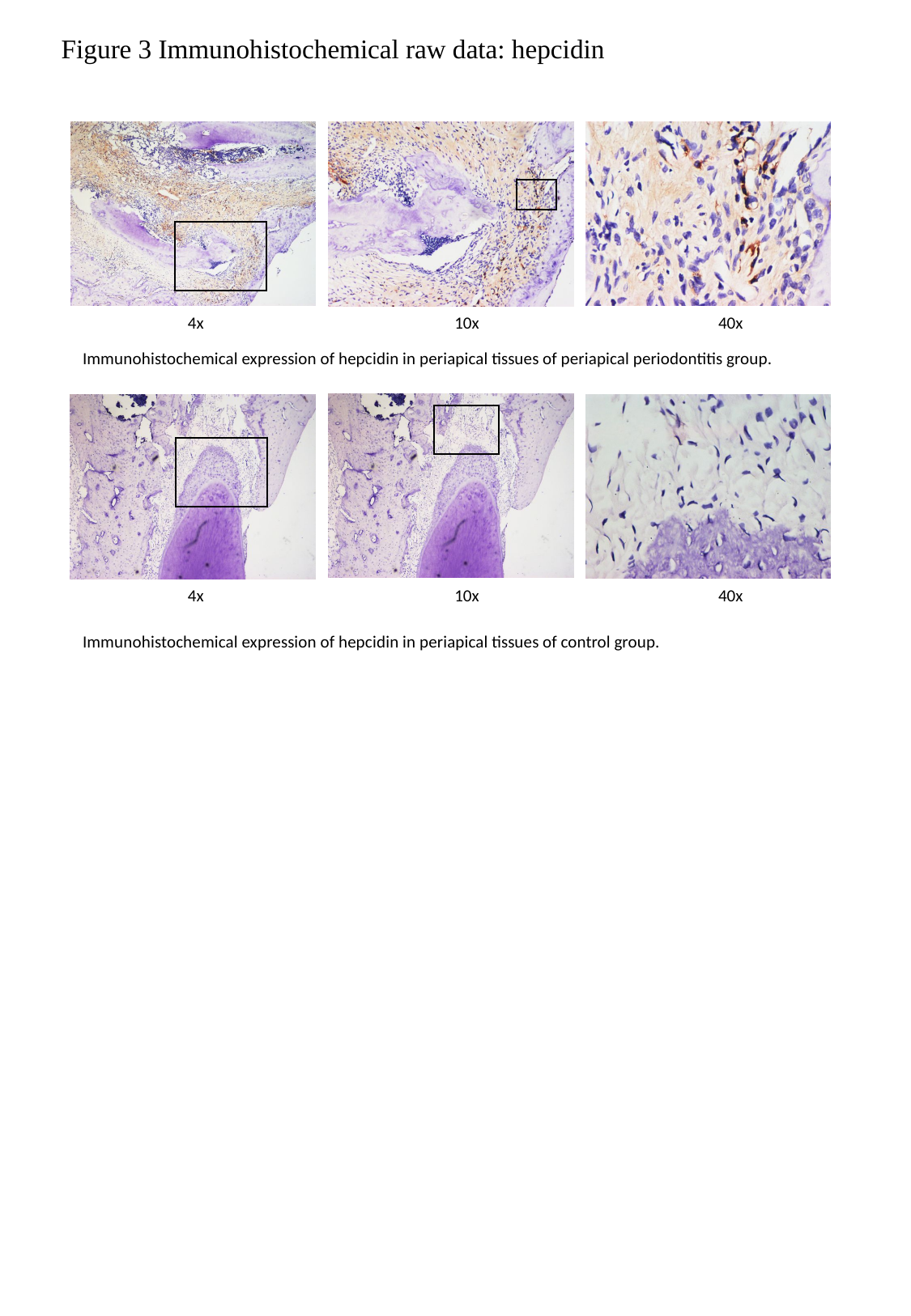

Figure 3 Immunohistochemical raw data: hepcidin
4x
10x
40x
Immunohistochemical expression of hepcidin in periapical tissues of periapical periodontitis group.
4x
10x
40x
Immunohistochemical expression of hepcidin in periapical tissues of control group.
