# Supplementary material for: Role of NOD2 and hepcidin in inflammatory periapical periodontitis
Source: BMC Oral Health. 2022 Jun 28;22:263. doi: 10.1186/s12903-022-02286-z (PMC9241313; doi:10.1186/s12903-022-02286-z)
Supplement: Supplementary file 2 — Additional file 2: Fig. S1 Top 30 significant enriched Gene Ontology (GO) including molecular function, cellular component and biological of 15 DEGs. [file 12903_2022_2286_MOESM2_ESM.pptx]

## Slide 1
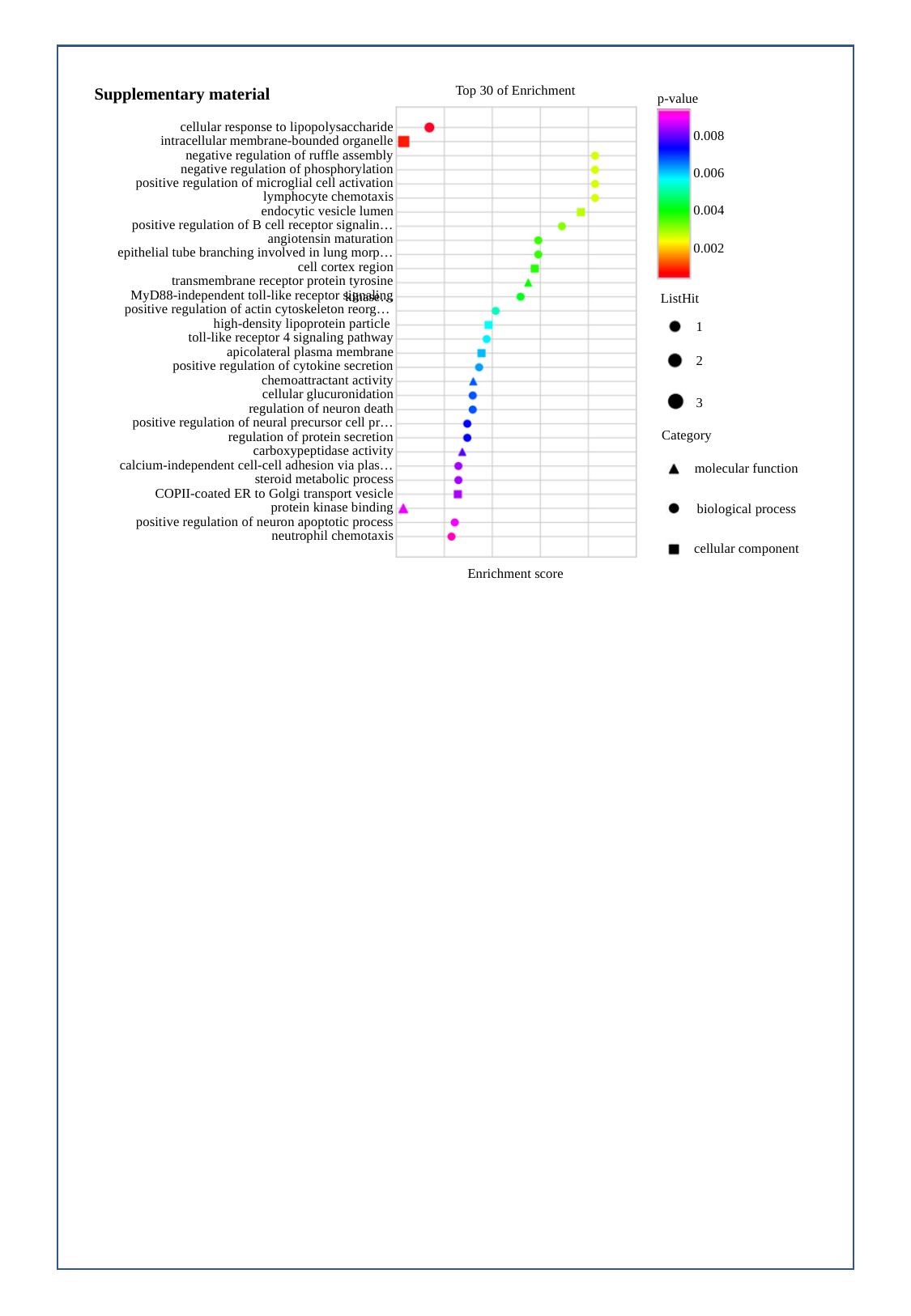

Top 30 of Enrichment
p-value
0.008
0.006
0.004
0.002
ListHit
1
2
3
Category
molecular function
biological process
cellular component
cellular response to lipopolysaccharide
intracellular membrane-bounded organelle
negative regulation of ruffle assembly
negative regulation of phosphorylation
positive regulation of microglial cell activation
lymphocyte chemotaxis
endocytic vesicle lumen
positive regulation of B cell receptor signalin…
angiotensin maturation
epithelial tube branching involved in lung morp…
cell cortex region
transmembrane receptor protein tyrosine kinase…
MyD88-independent toll-like receptor signaling
positive regulation of actin cytoskeleton reorg…
high-density lipoprotein particle
toll-like receptor 4 signaling pathway
apicolateral plasma membrane
positive regulation of cytokine secretion
chemoattractant activity
cellular glucuronidation
regulation of neuron death
positive regulation of neural precursor cell pr…
regulation of protein secretion
carboxypeptidase activity
calcium-independent cell-cell adhesion via plas…
steroid metabolic process
COPII-coated ER to Golgi transport vesicle
protein kinase binding
positive regulation of neuron apoptotic process
neutrophil chemotaxis
Enrichment score
Supplementary material
