# Supplementary material for: Role of NOD2 and hepcidin in inflammatory periapical periodontitis
Source: BMC Oral Health. 2022 Jun 28;22:263. doi: 10.1186/s12903-022-02286-z (PMC9241313; doi:10.1186/s12903-022-02286-z)
Supplement: Supplementary file 3 — Additional file 3. Gene expression values of STAP1, RPS4Y1, CTSZ, and UGT2B15 in inflamed and non-inflamed tissues obtained from GEO datasets. [file 12903_2022_2286_MOESM3_ESM.docx]

**Table S1** Gene expression values of STAP1, RPS4Y1, CTSZ and UGT2B15in inflamed and non-inflamed tissuesobtained from GEO datasets

|  | **inflamed tissue** | | | **uninflamed tissue** | | |
| --- | --- | --- | --- | --- | --- | --- |
|  | GSM282917 | GSM282922 | GSM282926 | GSM282867 | GSM282868 | GSM282869 |
| STAP1 | null | 2.298830348 | null | 1.092520234 | null | null |
| RPS4Y1 | 1.070476407 | 0.400729327 | 1.056472343 | 0.368490671 | 0.373867767 | 0.396996997 |
| CTSZ | 1.793776319 | 1.847955479 | 1.893112307 | 0.888048269 | 0.857239361 | 0.934537201 |
| UGT2B15 | 3.954512898 | 3.623791625 | 3.756268002 | 0.922122293 | 3.05273001 | 2.385679104 |
