# Supplementary material for: Role of NOD2 and hepcidin in inflammatory periapical periodontitis
Source: BMC Oral Health. 2022 Jun 28;22:263. doi: 10.1186/s12903-022-02286-z (PMC9241313; doi:10.1186/s12903-022-02286-z)
Supplement: Supplementary file 4 — Additional file 4. Gene expression values of CTSZ and CLDN8 in inflamed and non-inflamed tissues obtained from GEO datasets. [file 12903_2022_2286_MOESM4_ESM.docx]

**Table S2** Gene expression values of CTSZ and CLDN8 in inflamed and non-inflamed tissues obtained from GEO datasets

|  | **inflamed tissue** | | | **uninflamed tissue** | | |
| --- | --- | --- | --- | --- | --- | --- |
|  | GSM282917 | GSM282922 | GSM282926 | GSM282870 | GSM282871 | GSM282872 |
| CTSZ | 1.793776319 | 1.847955479 | 1.893112307 | 0.866889492 | 0.853267498 | 0.903514713 |
| CLDN8 | 3.520303952 | 3.05273001 | 3.366851289 | 0.958936374 | 2.191927377 | 1.15394143 |
