# Supplementary material for: Role of NOD2 and hepcidin in inflammatory periapical periodontitis
Source: BMC Oral Health. 2022 Jun 28;22:263. doi: 10.1186/s12903-022-02286-z (PMC9241313; doi:10.1186/s12903-022-02286-z)
Supplement: Supplementary file 5 — Additional file 5. Gene expression values of CXCL8, REG1A, S100A8, SAA1, TNIP3, MMP3, CXCL1, CXCL2, and CXCL3 in inflamed and non-inflamed tissues obtained from GEO datasets. [file 12903_2022_2286_MOESM5_ESM.docx]

**Table S3** Gene expression values of CXCL8,REG1A,S100A8, SAA1, TNIP3, MMP3, CXCL1, CXCL2 and CXCL3 in inflamed and non-inflamed tissues obtained from GEO datasets

|  | **inflamed tissue** | | | | | | **uninflamed tissue** | | | | | |
| --- | --- | --- | --- | --- | --- | --- | --- | --- | --- | --- | --- | --- |
|  | GSM282929 | GSM282933 | GSM282936 | GSM282937 | GSM282942 | GSM282945 | GSM282935 | GSM282938 | GSM282940 | GSM282941 | GSM282943 | GSM282944 |
| CXCL8 | 1.194270797 | 0.971190356 | 1.18304094 | 0.629915406 | 0.342671948 | 0.476946715 | 0.260255399 | 0.318485589 | 0.379165604 | 0.291102675 | 0.412852973 | 0.257706352 |
| REG1A | 2.615523985 | 2.555844387 | 2.03453627 | 3.310846213 | 1.030141129 | 2.357895858 | 1.086885526 | 1.043419283 | 1.009905289 | 1.031567338 | 1.075337852 | 1.060175422 |
| S100A8 | 2.71508996 | 2.452749672 | 2.878665185 | 2.424353371 | 0.663574338 | 1.120024274 | 0.89197798 | 0.674205359 | 0.909452378 | 1.030860418 | 0.761987903 | 0.662154599 |
| SAA1 | 2.46058269 | 3.07332428 | 2.558148474 | 3.628567229 | 0.989963336 | 2.339823975 | 0.755210119 | 0.744621924 | 1.016008168 | 0.714204931 | 2.069095163 | 0.837168538 |
| TNIP3 | 2.436820527 | 4 | 2.602142554 | 2.502551919 | 0.995668229 | 1.715822125 | null | 1.039971505 | 1.028852381 | 0.937348216 | 0.928195109 | 1.126252259 |
| MMP3 | 1.218857951 | 1.867696453 | 1.319215267 | 1.633334184 | 0.601172116 | 1.195115457 | 0.587377537 | 0.512686921 | 0.662039866 | 0.910057748 | 0.595395408 | 0.50326494 |
| CXCL1 | 3.304426714 | 4 | 3.555370725 | 2.891664147 | 1.122332384 | 1.862267088 | 0.919390714 | 0.875901169 | 0.811475813 | 0.911402353 | 0.768352373 | 1.232006613 |
| CXCL2 | 3.130081018 | 3.491144326 | 2.680124463 | 2.192383223 | 1.428498974 | 1.76377924 | 0.91944807 | 1.035699875 | 0.979587996 | 1.156367526 | 0.999257915 | 1.702116754 |
| CXCL3 | 1.930293199 | 2.020482702 | 1.889520276 | 1.638334565 | 0.931062551 | 1.451032668 | 0.600301842 | 0.70713619 | 0.718654374 | 0.759814948 | 0.593644028 | 1.214236966 |
